# NAVIGATING LIFE UNCOUPLED: CHANGES IN SINGLEHOOD SATISFACTION IN RESPONSE TO MAJOR LIFE EVENTS

**DOI:** 10.1093/geroni/igac059.123

**Published:** 2022-12-20

**Authors:** Jeewon Oh, William Chopik

**Affiliations:** Syracuse University, Syracuse, New York, United States; Michigan State University, East Lansing, Michigan, United States

## Abstract

Experiencing challenging events without a partner who can be supportive can be hard and change perceptions of the singlehood experience. We conducted growth curve analyses predicting intercepts and slopes of singlehood satisfaction from ten major life events (MLEs). Single people (N = 5,191; Mage= 46.98, 60.1% women) were followed longitudinally over 10 years (2008-2017) and provided information on MLEs and singlehood satisfaction. People were fairly satisfied with singlehood (Mintercept=6.52/10), and satisfaction increased over time (Mquadratic slope=.01). Some difficult events (e.g., becoming ill and losing parents) were associated with declines in singlehood satisfaction. However, responses to MLEs were overall complex—unemployment was related to lower levels of satisfaction but unrelated to changes in satisfaction. Seemingly positive events (e.g., buying a house, getting a job) were also related to lower levels of satisfaction. Results for each MLE will be discussed in line with additional qualitative data on single people’s perceptions of change.

